# Transformation of *Riccia fluitans*, an Amphibious Liverwort Dynamically Responding to Environmental Changes

**DOI:** 10.3390/ijms21155410

**Published:** 2020-07-29

**Authors:** Felix Althoff, Sabine Zachgo

**Affiliations:** Botany Department, School of Biology and Chemistry, Osnabrück University, 49076 Osnabrück, Germany; felix.althoff@uni-osnabrueck.de

**Keywords:** *Riccia fluitans*, liverwort, adaptation, terrestrialization, transformation, sexual induction

## Abstract

The colonization of land by streptophyte algae, ancestors of embryophyte plants, was a fundamental event in the history of life on earth. Bryophytes are early diversifying land plants that mark the transition from freshwater to terrestrial ecosystems. The amphibious liverwort *Riccia fluitans* can thrive in aquatic and terrestrial environments and thus represents an ideal organism to investigate this major transition. Therefore, we aimed to establish a transformation protocol for *R. fluitans* to make it amenable for genetic analyses. An *Agrobacterium* transformation procedure using *R. fluitans* callus tissue allows to generate stably transformed plants within 10 weeks. Furthermore, for comprehensive studies spanning all life stages, we demonstrate that the switch from vegetative to reproductive development can be induced by both flooding and poor nutrient availability. Interestingly, a single *R. fluitans* plant can consecutively adapt to different growth environments and forms distinctive and reversible features of the thallus, photosynthetically active tissue that is thus functionally similar to leaves of vascular plants. The morphological plasticity affecting vegetative growth, air pore formation, and rhizoid development realized by one genotype in response to two different environments makes *R. fluitans* ideal to study the adaptive molecular mechanisms enabling the colonialization of land by aquatic plants.

## 1. Introduction

The conquest of land by plants around 470 million years ago (MYA) formed new ecosystems as well as carbon cycles. This foundational event enabled the evolution of three-dimensional land plants with adaptations to new environments such that land plants represent today 80% of the total biomass on earth [1,2]. Recent molecular studies demonstrated that the monophyletic land plant group evolved from streptophyte algae and that the Zygnematophyceae, conjugating green algae, are sister to land plants [3,4,5]. The pioneering land colonization behavior of streptophyte algae is still present in modern habitats. *Zygnema* is a member of the advanced Zygnematales group of the streptophyte lineage that already possesses many genes required for a life on land. These streptophyte algae are abundant in freshwater as well as in hydro-terrestrial habitats and adapted to water loss and increased light exposure [6,7,8,9]. The earliest diverging land plants, the bryophytes, formed novel features to cope with the challenges associated with a permanent life on land. Breathing structures for CO_2_ uptake and balancing of water loss along with anchoring rooting structures mediating also nutrition and water uptake were key adaptations enabling to increase plant size and complexity during further land plant evolution [5,10]. The complex liverwort *Marchantia polymorpha* has been informative to understand the evolution of key adaptations such as rhizoid and air pore formation [11,12,13]. This dioecious plant forms for sexual reproduction gametangiophores, erected reproductive structures with antheridia and archegonia comprising the respective male and female gametes [14]. Appreciation of *M. polymorpha* for molecular analyses has been boosted by the availability of molecular tools, particularly different genome editing techniques for sporeling, gemmae, and thallus transformations [15,16,17,18,19,20]. *M. polymorpha* is a terrestrial species that is well adapted to growth on land. For investigation of the molecular mechanisms enabling the water-to-land transition, an ideal model would be a basal land plant species with the ability to thrive in freshwater as well as in terrestrial environments. This requires phenotypic plasticity, defined as changes in the phenotype realized by a single genotype in response to different environments [21].

*Riccia fluitans* is an amphibious liverwort that grows in water transition zones where it develops in dependence on water levels either a water form (WF) or a land form (LF) [22,23]. This widespread species, belonging to the subclass of Marchantiidae, quickly adapts to different environments by undergoing severe and traceable morphological changes that have intrigued scientists already since the early 20th century [24]. Further analyses investigated *R. fluitans* rhizoid and air pore formation in adaptation of the WF to the LF and recently molecular analyses started with sequencing of its chloroplast genome [22,25,26,27]. The phenotypic plasticity of *R. fluitans* enables dynamic adaptations of its morphogenesis to changing environments and reveals its potential to investigate molecular mechanisms contributing to the successful conquest of land.

Here, we established a transformation protocol for this amphibious liverwort facilitating functional genetic analyses and we demonstrate dynamic and reversible adaptations of *R. fluitans* plants to aquatic and terrestrial life styles. Abiotic stresses were investigated to induce a switch from vegetative to reproductive *R. fluitans* growth and two ecotypes were characterized that are available for future studies.

## 2. Results

### 2.1. Comparison of R. fluitans Water and Land Form Thalli

**Figure 1:**
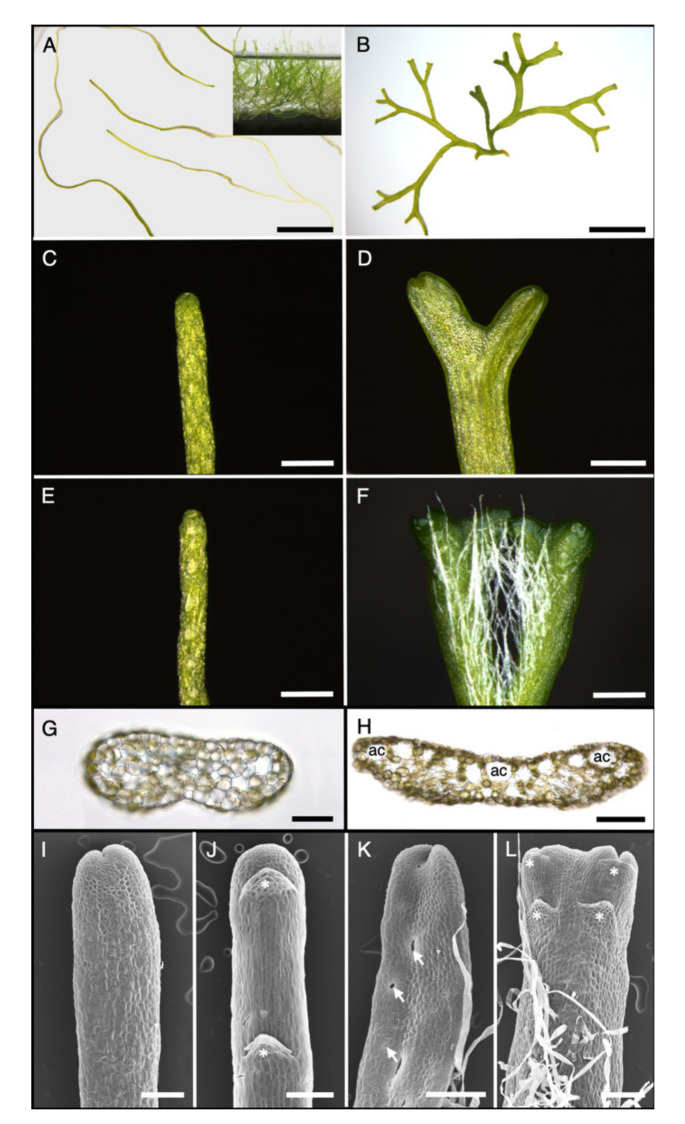
Land form (LF) and water form (WF) thallus morphologies of *Riccia fluitans* 001TC. (**A**) WF thalli were transferred to solid medium for image taking. Inset shows submersed growth of the WF. (**B**) LF thallus, grown on solid medium. (**C**) WF and (**D**) LF dorsal thallus side. (**E**) WF ventral thallus side. (**F**) LF ventral thallus side revealing thallus branching and rhizoid formation. (**G**) WF thallus cross section lacking air chambers. (**H**) LF cross section revealing air chambers (ac). (**I**–**L**) SEM pictures of thallus tips. (**I**) Dorsal WF thallus side lacking air pores. (**J**) Ventral WF side develops ventral scales (white asterisks) but no rhizoids. (**K**) Dorsal LF thallus side with air pores. Three air pores are indicated by white arrows. (**L**) Ventral LF thallus side forming rhizoids and ventral scales (white asterisks). Scale bars A,B: 5 mm; C–F: 500 µm; G–L: 200 µm.

The amphibious liverwort *R. fluitans* typically occurs as an aquatic species forming free floating mats and also grows as a terrestrial plant on moist grounds in drying ponds or streams [23]. *R. fluitans* is a popular aquarium plant and an axenic aquatic culture was purchased from an aquarium plant supplier and named *R. fluitans* 001TC according to the item number. *R. fluitans* 001TC was cultivated as WF (Figure 1A) and WF thallus pieces were spread on solid medium to induce growth of LF thalli (Figure 1B). 

Next, morphologies of the two *R. fluitans* 001TC forms were compared to identify distinct characteristic features. The WF develops slender, elongated thalli with a width of about 250–500 µm that branch only occasionally (Figure 1A,C) and grow towards the water surface (inset in Figure 1A). Ventral scales but no rhizoids are regularly formed on ventral WF thallus sides (Figure 1E,J). Cross sections reveal a continuous epidermal cell layer encompassing the WF thallus lacking air pores and air chambers for gas exchange. Cells containing chloroplasts are scattered throughout the epidermis and parenchymatous thallus (Figure 1G). LF thalli display different morphological features, where thallus width is increased to about 500–1000 µm and dichotomously branching occurs more frequently than observed in the WF (Figure 1B,D,F). Numerous rhizoids are formed on the ventral LF thallus side and, as in the WF, scales are regularly initiated (Figure 1F,L). Air pores in the LF epidermis and subjacent air chambers facilitate gas exchange (Figure 1H,K). The roundish or elongated pores are simple opening structures, lacking the complexity known from pores of other liverworts such as *M. polymorpha*. 

To summarize, *R. fluitans* 001TC forms under LF and WF growth conditions distinctive morphological features affecting thallus width, branching frequency, air pore, and air chamber formation for gas exchange as well as rhizoid development for anchoring. Our data support observations from earlier *R. fluitans* studies with other ecotypes [24,25,26], emphasizing that similar morphological adaptations of different ecotypes to two distinct growth environments enable molecular studies to unravel the underlying regulatory mechanisms. 

### 2.2. Induction of Reproductive Tissues 

The ability to induce the switch from vegetative to reproductive tissue formation is important for analysis of all *R. fluitans* life phases. It has been known for over 100 years that the *R. fluitans* WF easily propagates vegetatively via ruptured thallus pieces [24]. Sexual reproduction in bryophytes requires the formation of female and male gametangia, archegonia and antheridia, respectively. However, fertile *R. fluitans* producing archegonia as well as sporophytes have so far only been reported once and antheridia have never been observed [28]. Different approaches were tried to induce reproductive *R. fluitans* 001TC growth. At first, we tested the sexual induction treatment for the liverwort *M. polymorpha* [18] and grew both, the *R. fluitans* 001TC WF and LF, under long-day and also short-day conditions with far-red light supplementation. However, WF and LF plants continued vegetative growth even after more than 6 weeks of cultivation. Next, the impact of water availability was tested by transferring WF thalli to solid medium, where WF plants accordingly developed into the LF. After transfer of LF thalli to water, these floating plants were no longer anchored and developed WF features. Neither one of these transitions provided an appropriate stimulus to induce reproductive tissue formation. 

**Figure 2:**
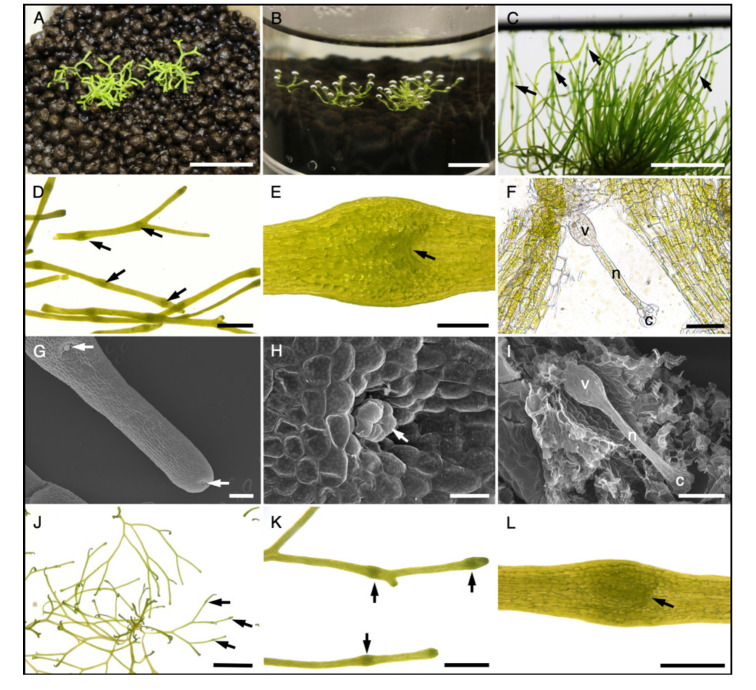
Reproductive tissue induction in *R. fluitans* 001TC by flooding and starvation. (**A**) Typical LF culture. (**B**) Culture shown in (A) 3 days after flooding with water, when thalli start to grow towards the water surface. (**C**) 14 days after flooding, the induced culture forms multiple archegonium cavities (arrows). (**D**) Close up of thalli with archegonium cavities (arrows). (**E**) Dorsal view of one archegonium cavity with entrance marked by an arrow. (**F**) Squash mount of an archegonium cavity to visualize the archegonium. Venter (v), neck (n), cover cells (c) of the archegonium. (**G**–**I**) SEM pictures. (**G**) Dorsal side of induced thallus with two archegonium cavities (arrows), the youngest is initiated close to the meristematic zone. (**H**) Archegonium cover cells (arrow) protruding the archegonium cavity. (**I**) Removal of dorsal thallus tissue uncovers the archegonium in the archegonium cavity. (**J**–**L**) *R. fluitans* 001TC forms archegonium cavities (arrows) 5 weeks after cultivation on solid 1/100 GB5 starvation medium. (**K**) Close up of archegonium cavities (arrows). (**L**) Archegonium cavity with entrance (arrow). Scale bars A,B,C,J: 1 cm; D, K: 2 mm; E,L: 500 µm; F,I: 100 µm; G: 200 µm; H: 50 µm.

Considering natural *R. fluitans* habitat conditions, flooding of LF thalli adhering on the ground can occur after strong rainfalls and could mediate inductive conditions. A LF culture was flooded with water to simulate raising water levels in ponds (Figure 2A,B) and their thalli did not drift to the water surface, as they were still anchored to the substrate by their rhizoids (Figure 2B). 

Plants reacted to this stimulus after around 3 days by directing thallus growth towards the water surface (Figure 2B). Notably, after around 7 days, plants started to produce morphological changes, discernable as thallus swellings. After 14 days of submersed thallus growth, several swellings were regularly spaced every 5–10 mm along the thalli (Figure 2C,D). Closer inspection revealed that these thallus areas represent archegonium cavities, which contain a single archegonium with a length of about 300 µm (Figure 2E,F,I). *Riccia* archegonia are composed of a central egg cell enfolded by venter cells. Neck cells form a tubular structure ending with cover cells at the tip that are reaching out of the archegonium cavity (Figure 2F,H,I) [29]. Cover cells of the youngest archegonium are already visible close to the apical cells, indicating that archegonia formation is initiated in the meristematic region at the thallus tip (Figure 2G). Induction of female reproductive growth was reproduced several times independently, providing the robustness of the anchored LF-WF *R. fluitans* sexual induction system.

Selkirk [30] reported an induction of *R. fluitans* archegonia under nutrient starvation conditions. To test this condition, we transferred LF thalli to liquid and to solid starvation medium containing only 1/100 GB5. The plants on solid starvation medium developed elongated, yellowish thalli within a few days indicating nitrogen deficiency. After 5 weeks, first thallus swellings with archegonium cavity formation were observed (Figure 2J–L). Plants transferred to 1/100 GB5 liquid medium floated on the medium, likely mediated by air chambers of LF thalli. These plants remained in the vegetative phase, decreased growth, and ultimately died. Reproductive *R. fluitans* tissues have only very rarely been documented in the wild [28] and our analyses in the natural habitat of the Botanical Garden of Osnabrück support the assumption that *R. fluitans* reproduces mainly vegetatively. 

Here, we report a reliable induction of female reproductive growth within 2–3 weeks by flooding LF thalli with water. Additionally, induction of *R. fluitans* archegonia formation is also possible by transferring LF thallus to a solid starvation medium, which then takes 5 weeks.

### 2.3. Analysis of Dynamic Growth Transitions in Individual R. fluitans Plants

**Figure 3:**
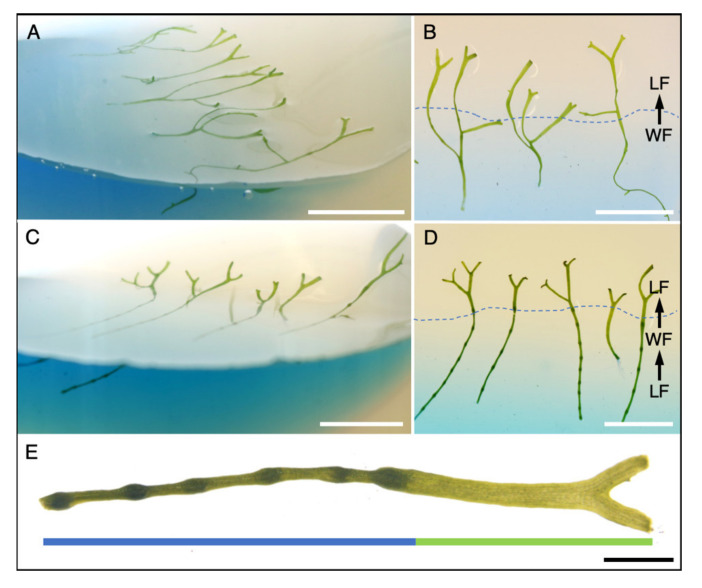
Phenotypic plasticity of *R. fluitans* 001TC plants. (**A**) WF thalli after growing for 13 days in a growth container with a slanted agar surface, liquid medium is stained with toluidine blue. WF thallus tips entered the solid phase and developed branching LF thalli. (**B**) Top view of (A) with a dashed line indicating the water/land border. (**C**) A LF culture was flooded to induce WF and archegonia formation and was then transferred into a slanted agar growth container and grown for 17 days. WF thallus tips entered the solid phase, stopped producing archegonium cavities and developed LF features, such as thallus branching. (**D**) Top view of (C). (**E**) Visualization of sequentially formed, distinct morphological adaptations within a single plant that has experienced LF–WF–LF transitions. The blue bar marks sexual WF thallus induced by flooding of the LF and the green bar indicates formation of the broadened, typical LF thallus with dichotomous branching. Scale bars A–D: 1 cm; E: 1 mm.

We further investigated the adaptive morphological transformations occurring within individual plants during the switch from water to land under in vitro culture conditions. To mimic a shore situation with a water-to-land transition zone, growth containers with slanted agar surfaces were prepared and filled with liquid medium. Thalli from a WF culture were transferred to the water part of a slanted agar container. When growing WF thalli reached the imitated transition zone, their tips came into contact with agar. In response to the changed environment, thalli broadened and started to branch regularly, revealing typical LF features including rhizoid formation for anchorage on the solid medium (Figure 3A,B). 

Next, we investigated the response of sexually induced thalli to cultivation on land by conducting double LF–WF–LF transition experiments. Archegonia formation was induced in LF thalli by water flooding and thalli harboring archegonium cavities were then transferred to the liquid part of slanted agar containers. When these thalli reached the solid phase, plants quickly adopted to the novel environment imitating the shore zone of a pond or stream. We then observed a broadening of the thallus, initiation of thallus branching, and thalli stopped producing further archegonium cavities (Figure 3C,D). 

These data demonstrate that single *R. fluitans* plants are capable to respond consecutively to the LF–WF–LF transition conditions, which enables to observe dynamic vegetative and reproductive growth responses of one genotype to two different environments along the growth axis of a single plant (Figure 3E). 

### 2.4. Establishment of a Transformation Protocol for R. fluitans

Spore, gemmae, and thallus transformation protocols have been established for the related liverwort species *M. polymorpha* [18,19,20]. Given the absence of gemmae production in *R. fluitans* and lack of a male line for spore generation, we aimed to establish a *R. fluitans* thallus transformation protocol. 

At the start, four antibiotics frequently used for *M. polymorpha* selection were tested for *R. fluitans* 001TC. LF thallus was chopped and spread on medium containing hygromycin (2.5, 5, 10 µg/mL), G418 (5, 10, 20 µg/mL), chlorsulfuron (0.25, 0.5, 1 µM), and gentamycin (100, 200, 400 µg/mL) (Table S1). Observation of mortality rates after 1, 2, and 3 weeks identified 5–10 µg/mL hygromycin and 20 µg/mL G418 as suitable selections, whereas chlorsulfuron and gentamycin were not effective.

The binary pMpGWB-mCherry vector was used for establishing a transformation protocol. This pGWB2 derivate vector comprises the *hpt* gene mediating hygromycin resistance and the mCherry CDS, which was used as reporter fluorophore expressed under the control of the *M. polymorpha* Mp*EF1α* promoter [31,32]. At first, a method based on *M. polymorpha* transformation protocols [18] was tested using chopped *R. fluitans* 001TC LF thallus pieces, which failed to produce lines surviving the hygromycin selection. Several modifications such as injuring thallus pieces in a bead ruptor, enzymatic digestion of cell walls and testing different *Agrobacterium* strains (EHA105, LBA4404, GV3101) as well as co-cultivation on solid medium and in the dark, reported to increase *M. polymorpha* transformation efficiencies [33], also failed to generate hygromycin-resistant *R. fluitans* lines expressing mCherry. 

**Figure 4:**
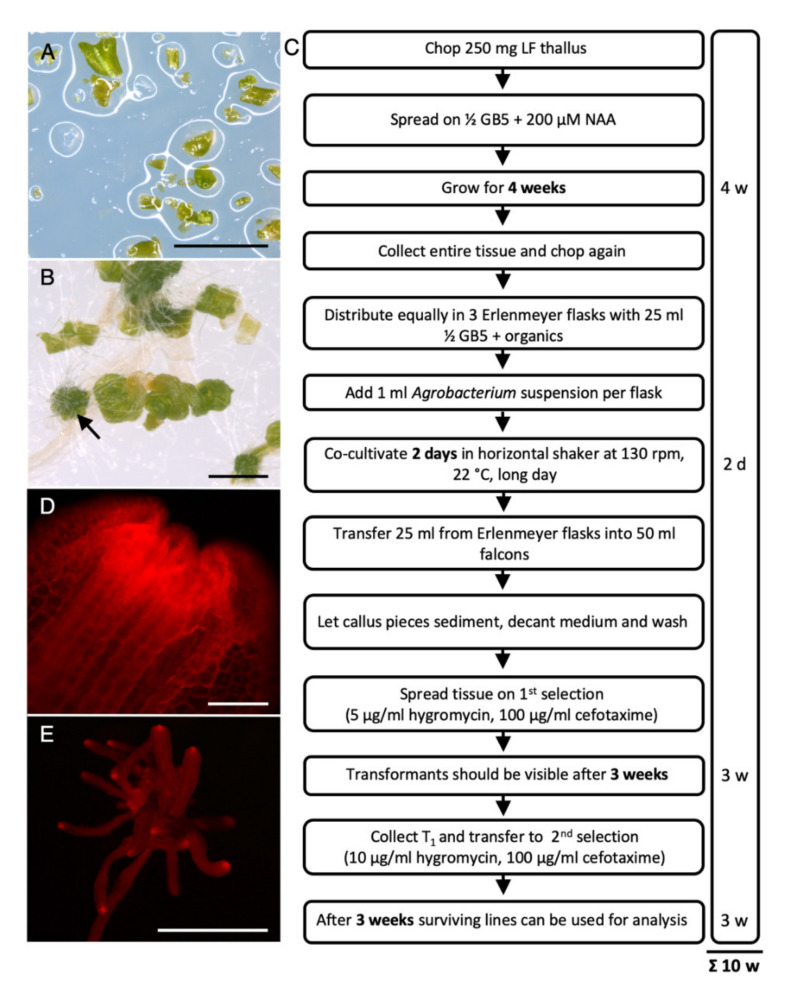
Scheme of the *R. fluitans* 001TC transformation procedure. (**A**) Chopped LF thallus pieces were plated on 200 µM NAA-containing medium. (**B**) Four weeks after plating NAA induced mainly callus (arrow) and also curled thallus tissue growth. (**C**) Overview of the transformation procedure. (**D**) mCherry expression in thallus tip region of a *_pro_*Mp*EF1α*::*mCherry* T1 line. (**E**) mCherry signal detection in a transgenic line after three rounds of vegetative propagation. Scale bars A,B: 2 mm; D: 5 mm; E: 100 µm.

To optimize transformation approaches for species that are difficult to transform, such as rice and maize, callus tissue has been successfully used [34,35]. Formation of callus tissue, comprising redifferentiated, pluripotent cells can be induced by auxin application [36]. Addition of 10 µM 1-naphthaleneacetic acid (NAA), a synthetic auxin, has been reported to affect growth of *M. polymorpha*, but was not sufficient to induce callus growth [37]. To determine a NAA concentration suitable to induce callus formation in *R. fluitans*, LF thalli were chopped into 1 mm pieces (Figure 4A) and spread on medium containing NAA concentrations of 50, 100, 200, and 400 µM. The 50 and 100 µM NAA caused only curling of the thalli and 400 µM NAA exerted a severe growth inhibiting effect causing early tissue death. Growth on 200 µM NAA was ideal, as it led to callus tissue formation of most tissue pieces and only a few pieces developed into small and curled thallus-like structures (Figure 4B).

Therefore, 250 mg chopped *R. fluitans* LF thallus pieces were plated on a 200 µM 1-naphthaleneacetic acid (NAA) plate. After 4 weeks of cultivation, all tissues were collected from the plate, chopped again to 1 mm pieces, and equally distributed into three Erlenmeyer flasks containing 25 mL liquid ½ GB5 medium. The three cultures were each inoculated with 1 mL *Agrobacterium* cells harboring the pMpGWB-mCherry vector and further grown for 2 days. After removal of *Agrobacterium* cells, plant tissue from each Erlenmeyer flask was distributed on three selection plates (Figure 4C). Three independent experiments were carried out, each starting with 250 mg chopped thallus used for generating one callus plate per experiment. Concentration of 5 and 10 µg/mL hygromycin were used for selection and also 2.5 µg/mL hygromycin was tested as calli might be more sensitive to hygromycin than the investigated thallus tissue.

Selection with 2.5 µg/mL hygromycin resulted in growth of 144 surviving candidates that were transferred to a second selection step using 10 µg/mL hygromycin. Seven transformants survived the second selection, all of which expressed mCherry fluorescence (Table 1, Figure 4D). Using a 5 µg/mL hygromycin selection, the number of surviving lines was lower, and 39 lines could be transferred to plates with 10 µg/mL hygromycin. Of these, five lines survived and revealed mCherry fluorescence (Table 1). Only one plant survived the first selection on 10 µg/mL hygromycin, which also continued growth under the second 10 µg/mL hygromycin selection and showed mCherry signal (Table 1). 

**Figure 5:**
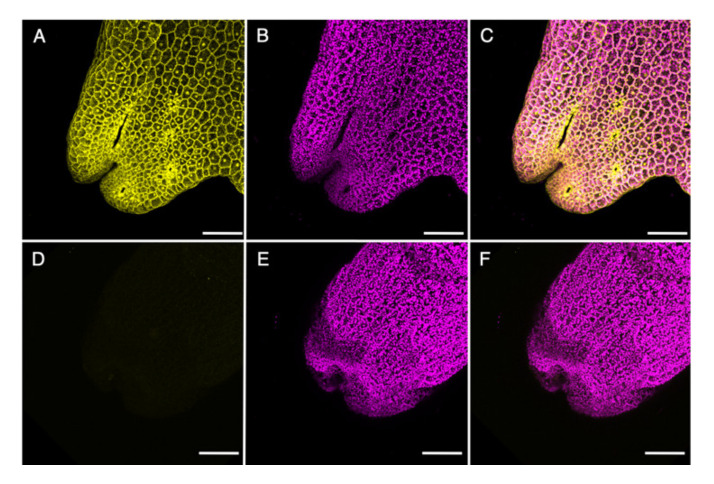
Transformed *R. fluitans* 001TC plant expressing mCherry. (**A**–**C**) *_pro_*Mp*EF1α*::*mCherry* T1 line. (**D**–**F**) Wild type control. (**A**,**D**) mCherry fluorescence channel. (**B**,**E**) Chlorophyll a autofluorescence channel. (**C**) Merged channels of (A) and (B). (**F**) Merged channels of (D) and (E). Scale bars 200 µm.

Strong mCherry expression was observed in cells of the thallus tips and further subcellular investigation detected mCherry fluorescence in the cytoplasm and nuclei of these meristematic cells (Figure 5A). The mCherry signals did not overlap with chlorophyll a autofluorescence (Figure 5B,C) and no signal could be detected in untransformed wild type controls (Figure 5D–F).

The stability of the transformations was tested by chopping thalli from three lines (*_pro_*Mp*EF1α*::*mCherry^#1^*, *_pro_*Mp*EF1α*::*mCherry^#4^*, *_pro_*Mp*EF1α*::*mCherry^#5^*) and regenerating growth on solid medium with 10 µg/mL hygromycin selection. After three consecutive repetitions of this vegetative propagation procedure, regenerated plants of the three lines were still expressing mCherry in meristematic regions of their regenerating thalli (Figure 4E). Additionally, thalli of the third propagation step from the three lines and from one wild type control line were chopped and again spread on solid medium without selection. After recovery of thallus growth for 4 weeks, 60 young plants per line were again transferred to selection medium containing 10 µg/mL hygromycin. All 60 plants of the three transgenic lines continued normal thallus LF growth, while the control wild type plants died (Figure S1). These data demonstrate that the hygromycin and mCherry expression remained stable throughout prolonged cultivation times and over four vegetatively propagated generations.

To summarize, we established a *R. fluitans* callus transformation protocol enabling to generate stably transformed T1 lines within 10 weeks. In three transformation experiments 13 independent lines were generated, which showed all fluorophore expression and revealed under selective conditions a wild type like growth. 

### 2.5. Characterization of Two R. fluitans Ecotypes 

**Figure 6:**
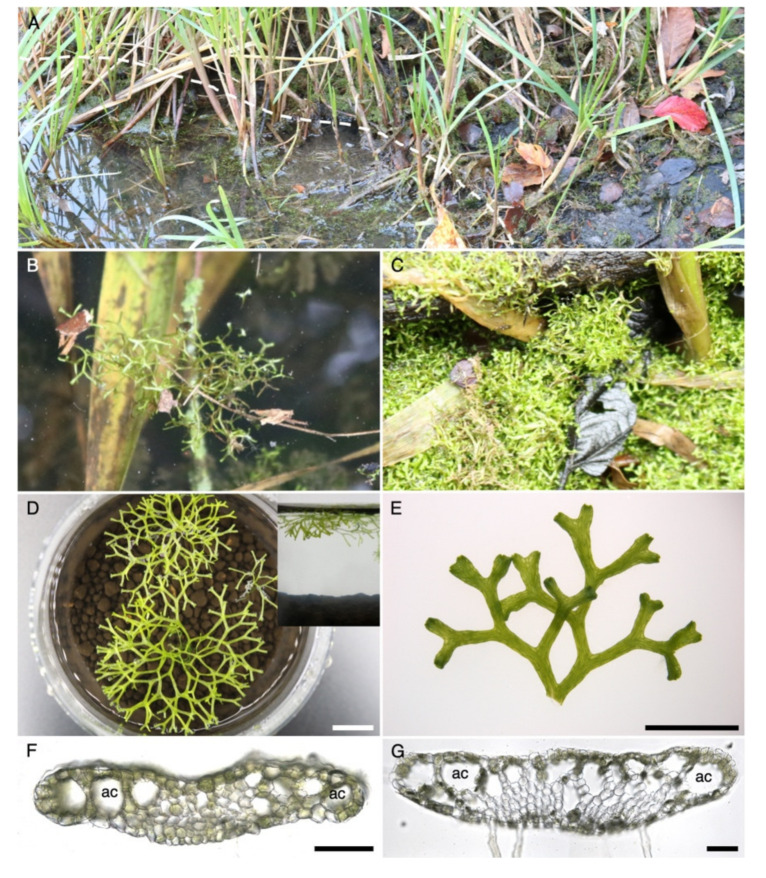
*R. fluitans* BoGa ecotype. (**A**) *R. fluitans* BoGa in its natural habitat, a waterside of the main pond in the Botanical Garden of Osnabrück. White dashed line indicates the water to land border. (**B**) Floating WF thalli of *R. fluitans* BoGa. (**C**) LF forms dense mats close to the littoral zone. (**D**) Top view of a WF culture, where inset shows a side view of the floating culture. (**E**) LF grown in axenic culture. Thallus cross section with air chambers (ac) of the WF (**F**) and LF (**G**). Scale bars D: 1 cm; E: 5 mm; F, G: 100 µm.

High inter-species similarities exist within the *Riccia* genus. Furthermore, *R. fluitans* is difficult to be distinguished within the taxonomically challenging *R. fluitans* complex, which is further complicated by the two morphologically distinct *R. fluitans* LF and WF. These obstacles can lead to misidentifications and even *R. fluitans* LF and WF have been described as different species [22,38]. In addition to *R. fluitans* 001TC with an unknown origin, we established an axenic culture of an ecotype derived from the main pond of the Botanical Garden of the University of Osnabrück and named this ecotype *R. fluitans* BoGa. In this habitat, plants grow free floating in the water (Figure 6A lower side of white dashed line, Figure 6B) as well as terrestrial, close to the littoral zone (Figure 6A upper side of dashed line, Figure 6C).

Further analyses were conducted to exclude species misinterpretations. The *R. fluitans* LF can be mixed-up with *R. canaliculata*, forming as distinctive features a furrowed thallus and an apical scale overtopping the apex [39] not observed in BoGa and 001TC ecotypes (Figure 1J,L). The *R. fluitans* WF resembles *R. stenophylla,* which is monoecious and forms consistently sporophytes [23], which were not formed by both *R. fluitans* ecotypes. Moreover, the WF of *R. fluitans* and *R. rhenana* are morphologically highly similar. Univocal discrimination is possible by chromosome counting, as *R. fluitans* possesses eight and *R. rhenana* 16 chromosomes [23]. Chromosome analysis of *R. fluitans* BoGa and *R. fluitans* 001TC revealed that both ecotypes do not possess duplicated chromosome sets (Figure S2). 

The *R. fluitans* BoGa WF floats just below the water surface, while the *R. fluitans* 001TC WF exhibits a more submersed growth (Figure 1A and Figure 6D). Cross sections revealed that the *R. fluitans* BoGa WF develops air chambers, which likely mediate an impetus towards the water surface (Figure 6F), whereas the WF of the *R. fluitans* 001TC ecotype does not form air chambers, explaining a submersed growth (Figure 1G). LF comparisons showed that the *R. fluitans* BoGa LF forms slightly larger air chambers compared to *R. fluitans* 001TC (Figure 6G). 

Taken together, morphological and chromosome analyses demonstrate a clear *R. fluitans* species affiliation for the two investigated different ecotypes, which can be used for future transformation approaches. 

## 3. Discussion

The liverwort *M. polymorpha* is well adapted to a terrestrial life and has been established as a basal land plant model system [14,40,41]. Notably, liverworts also comprise the amphibious species *R. fluitans*, which lives in water and on land and is thus ideal to close the gap between aquatic and terrestrial plant model species. Here, we report the establishment of *R. fluitans* transformation and sexual induction protocols and characterized dynamic growth adaptations.

### 3.1. Agrobacterium Mediated Transformation Makes R. fluitans Amenable for Molecular Analyses 

Given the absence of gemmae and spore formation in *R. fluitans*, various transformation approaches using thallus pieces were initially tested that were not successful. For optimization, we tried callus tissue, a cell mass of redifferentiated pluripotent cells known to mediate efficient and stable transformations in maize and rice [34,35,42,43]. Application of callus tissue enabled the successful transformation of *R. fluitans* and 13 transgenic lines were generated in three experiments. The transformation procedure starts with the induction of callus growth from LF thalli followed by *Agrobacterium* co-cultivation and two consecutive selection steps, together generating transgenic *R. fluitans* lines in 10 weeks. 

We conclude from our tested conditions that a first selection with 5 µg/mL and a second selection with 10 µg/mL hygromycin is advisable to increase transformation results. It has been shown that the Mp*EF1α* promoter drives strongest reporter gene expression in meristematic zones of *M. polymorpha* [32], which was similarly observed in transformed *R. fluitan*s plants. The 13 transgenic *R. fluitans* lines showed strongest mCherry fluorophore signals in thallus tips. Three lines were further investigated and survived three successive vegetative propagation steps under selection and continuously expressed mCherry. After an additional cultivation for 4 weeks without selection followed by growth on selection plates, again all propagated lines survived and expressed mCherry. 

Together, these alternating selection regimes over prolonged cultivation periods strongly support that continuous expression of the hygromycin resistance and fluorophore genes are the result of stable transformations. 

### 3.2. Abiotic Stress Stimuli Induce R. fluitans Reproductive Tissue Formation

As sessile organisms, plants are exposed to various stresses from which they cannot escape and thus require developmental and metabolic adaptations. Starvation is an important stimulus to switch from vegetative to reproductive growth in chlorophytic algae as well as in angiosperms, benefiting from recombination events to adapt to novel environmental conditions [44,45]. A report that *R. fluitans* archegonia formation is induced under starvation conditions [30] prompted us to test our investigated ecotypes. *R. fluitans* 001TC and BoGa LF thalli that were cultivated on 1/100 GB5 medium developed archegonia after 5 weeks. 

Additionally, we investigated whether flooding induces *R. fluitans* to enter the reproductive phase. Flooding is a major environmental change that is likely often experienced by the *R. fluitans* LF and requires rapid metabolic and morphological adaptations. We observed that thalli of flooded LF plants quickly developed the WF and initiated archegonia after only 2 weeks. Interestingly, under both stress conditions, starvation and flooding, induced *R. fluitans* thalli revealed a similar morphology and formed thin, elongated, less branched thalli with a brighter greenish coloration. The thallus stress-response phenotype was formed independently of aquatic or terrestrial growth and archegonia emerged on an induced WF and also on a starving LF. The two independent induction systems demonstrate that different abiotic stresses can cause an induction of reproductive tissues in *R. fluitans*. 

Furthermore, we demonstrate for the first time that a single *R. fluitans* plant can switch from vegetative to reproductive growth and then back to vegetative growth, revealing a remarkable capability to adapt dynamically to different environments. Notably, Hellwege and colleagues showed that abscisic acid (ABA) levels are increasing in *R. fluitans* WF plants transforming into LF plants and that WF to LF transition can be induced by supplementation of ABA in an aquatic environment [26]. Charophyceae, freshwater green algae*,* possess homologs of ABA synthesis but lack the genes for the receptors [46], whereas liverworts already possess all components of the ABA signaling pathway [41]. Functional ABA signaling might therefore have exerted an important contribution to land plant adaptations.

Thus far, only one fertile *R. fluitans* population with archegonia and sporophytes has been reported, however without any male structure identification [28]. Donaghy [24] described an additional sporophyte formation in *R. fluitans* LF plants, but as he mentioned a furrowed thallus, the described plants could have been *R. canaliculata* and not *R. fluitans*. It is known that misdeterminations can occur in the *Riccia* genus that are caused by high inter-species similarities and also intra-species variations considering the different WF and LF morphologies of *R. fluitans* [22,38]. Hence, in addition to morphological analyses to discriminate the two investigated *R. fluitans* ecotypes from highly similar species*,* chromosome counting was carried out. Thereby, we demonstrate that *R. fluitans* 001TC and *R. fluitans* BoGa were correctly identified. Although antheridia formation in *R. fluitans* has not been reported, it is commonly accepted that *R. fluitans* is a dioecious species [23,39]. The *Riccia* genus contains dioecious and monoecious species [39] supporting that both possibilities could hold true. In our studies, we observed that *R. fluitans* 001TC forms female structures and also all isolated and induced *R. fluitans* BoGa plants only formed archegonia. This invariable gender occurrence suggests that each ecotype could have been formed by clonal reproduction of a single female individual. In our Botanical Garden, similar to what McGregor [47] observed, a thallus fragment might have been introduced by a water bird followed by further spreading throughout the pond. The two established sexual induction approaches will be helpful to further investigate whether amphibious *R. fluitans* forms female and male structures and whether this occurs on the same or on distinct plants.

### 3.3. Adaptations to the Two R. fluitans Life Forms and Ecotype Differentiations

In comparison to *M. polymorpha* that forms smooth and pegged rhizoids, *R. fluitans* only forms smooth rhizoids as pegged rhizoids were secondarily lost in this genus [48]. Air pores and air chambers are crucial structures for gas exchange and respiration. In *Riccia*, air pores are simple openings, while air pores in *Marchantia* are complex, forming a compound barrel shape [14]. Molecular data indicate that compound, complex air pores are ancestral in liverworts and that the simple pores formed by *Riccia* are derived [48]. Together, secondary losses of pegged rhizoids and complex air pores indicate that *Riccia* holds a derived and not basal phylogenetic position in the Marchantiidae sub-class [48,49].

Interestingly, most liverwort lineages have notably slow rates of evolution, including *Marchantia*, whereas *Riccia* reveals a faster evolution rate and is the most species-rich genus in complex liverworts [48,50]. The faster evolution rate might have supported the evolution of its phenotypic plasticity and ability to grow submersed, which is likely rather a secondary adaptation of *R. fluitans* given the exceptional amphibious lifestyle of *R. fluitans* within the *Riccia* genus. Additionally, it might also have supported further adaptations to different ecological niches. *R. fluitans* 001TC, with an unknown origin, might have evolved submersed growth in adaption to special light and CO_2_ concentrations [51] and could therefore have been selected to meet the demands of customers using this liverwort for greening of aquarium bottoms. We generated a novel axenic *R. fluitans* BoGa ecotype culture with known origin and compared it with the *R. fluitans* 001TC ecotype. While *R. fluitans* 001TC WF lacks air chambers which likely mediate a submersed growth, BoGa ecotype WF thalli develop air chambers and float below the water surface. These minor morphological differences could be adaptations to individual ecotype habitats with varying light availability, as floating forms might inhabit deeper, turbid lakes whereas submersed growing forms inhabit shallow, clear waters. It has been reported that *R. fluitans* can sink to the bottom of ponds to survive colder temperatures during winter time [24,28], which further strengthens an adaptability by likely adjusting air chamber formation and thus thallus impetus to altered environmental conditions.

### 3.4. Outlook

The amphibious life style of *R. fluitans* liverwort enables the investigation of dynamic transitions between water and land plant forms as well as adaptations to different stress factors in a single basal land plant. Two ecotypes, *R. fluitans* 001TC and *R. fluitans* BoGa, are available as axenic cultures and the establishment of transformation and sexual induction protocols make *R. fluitans* accessible for future molecular analyses. Drought, UV light, and strongly fluctuating temperatures have been central abiotic stressors for plants during the conquest of land over 470 MYA until today, where climate change represents a challenge. Investigating the impact of these diverse stresses on *R. fluitans* growth and unravelling the adaptive molecular mechanisms will provide insights in plant terrestrialization processes and support climate-change focused plant breeding.

## 4. Materials and Methods 

### 4.1. Plants and Growth Conditions

An axenic in vitro culture of *R. fluitans* was purchased from Tropica Aquarium Plants (Egå, Denmark) (https://tropica.com/en/plants/plantdetails/Ricciafluitans(001TC)/4386) and accordingly named *R. fluitans* 001TC. For LF growth *R. fluitans* 001TC was cultivated on damp autoclaved aquarium scaper’s soil (Dennerle GmbH, Münchweiler an der Rodalb, Germany) and WF thalli were grown in containers with a bottom layer of scaper’s soil filled with ddH_2_O. For vitro cultivation, LF was grown on solid half strength Gamborg’s B5 medium including vitamins (½ GB5) (Duchefa, Haarlem, The Netherlands) supplemented with 14 g/L agar-agar Kobe I (Roth, Karlsruhe, Germany). WF thalli were grown on solid ½ GB5 medium overlaid with liquid ½ GB5 medium. 

Slanted growth containers were prepared by fixing cylindrical growth containers at a 45° angle while the agar solidified, which was afterwards overlaid with liquid ½ GB5 medium. Induction of reproductive tissues by flooding was carried out axenically on agar or on aquarium scaper’s soil. Antibiotics for selection of transgenic lines were added at indicated concentrations. Plants were grown in a climate chamber at 22 °C under long day conditions (16 h light:8 h dark) and cool white (840) fluorescent tubes emitting 90 µmol m^−2^ s^−1^ photons. 

*R. fluitans* thalli were also collected from the main pond of the Botanical Garden of the University of Osnabrück, referred to as *R. fluitans* BoGa. Tips from WF thalli were isolated and rinsed under running water for 15 min to remove debris. Tissues were then surface sterilized using 50 µL NaOCl (11–15% available chlorine) in 1 mL ddH_2_0 for 4 min and afterwards washed with ddH_2_O. Sterilized thalli were transferred to solid ½ GB5 plates containing 100 µg/mL cefotaxime (Duchefa, Haarlem, The Netherlands) for removal of remaining bacteria. 

Voucher specimens are deposited at the OSBU herbarium under accession numbers OSBU 28206 (*R. fluitans* BoGa) and OSBU 28207 (*R. fluitans* 001TC).

### 4.2. Microscopy and Sectioning

Habitus pictures were taken with a Canon EOS 750D DSLR camera or Leica M165 FC stereo microscope equipped with a Leica DFC490 camera. Pictures of cross sections, archegonia, and chromosome analyses were taken with a Leica DM5000 B microscope and Leica DFC490 camera. 

80 to 130 µm thallus cross sections were prepared using a Leica VT1000S vibratome. Thalli were immersed in 1% liquid agarose and vacuum was applied for 30 s. Degassed thalli were transferred to 3% liquid agarose and hardened by cooling to room temperature. 

Tissues for scanning electron microscopy were fixed in FAE fixative (2% formaldehyde, 5% acetic acid, 50% ethanol) at 4 °C for 2 days and then washed and dehydrated using an ethanol series. Critical point drying was conducted in the Leica EM CPD300 system. The chamber was cooled to 10 °C, 36 CO_2_ changes were applied to the tissue, and the chamber was afterwards heated to 40 °C. Dried samples were gold sputtered in the Leica EM ACE600 sputter coater. Sputtering was performed twice at 0° and 15° angles resulting in a gold coat of 10 nm. Samples were analyzed with a Jeol JSM-IT200 scanning microscope. 

Fluorescence pictures of transgenic thalli were taken with a Zeiss LSM 880 in airy scan mode. For mCherry detection, thalli were excited with laser DPSS 561-10 at a wavelength of 561 nm and maximum emission was detected at 615 nm. Chlorophyll a autofluorescence was exited at 633 nm using the laser HeNe633 and maximum emission was detected at 687 nm. Raw pictures were processed and assembled using the Fiji image processing program (version 2.0.0-rc-69/1.52i) [52]. 

### 4.3. Chromosome Counting

Chromosome counting was carried out according to Schwarzacher [53]. Briefly, thallus tips from freshly started *R. fluitans* 001TC and *R. fluitans* BoGa cultures ensuring high cell division rates, were collected and transferred to ice water for 24 h and afterwards incubated in saturated 8-hydroxyquinoline solution for 3 h. Thallus pieces were then fixed overnight in Carnoy’s solution. The next day, thalli were washed five times with 10 mM citric acid buffer (pH 4.6) to remove the fixative. Thalli were then digested with 2.5% pectolyase (Duchefa, Haarlem, The Netherlands), 2.5% pectinase (Sigma-Aldrich, Darmstadt, Germany), and 2.5% cellulase (Duchefa, Haarlem, The Netherlands) for 30 min and washed with ddH_2_O. DAPI (10 µg/mL) staining was performed on object slides, tissue was then squashed with a cover slip, and staining results were immediately observed using a Leica DM5000 B microscope.

### 4.4. Generation of R. fluitans Callus Tissue and Transformation Procedure

First, 250 mg of axenic grown LF thallus was chopped into 1 mm pieces with a flame-sterilized razor blade and spread with a Drigalski glass spatula on one ½ GB5 plate supplemented with 200 µM NAA (Duchefa, Haarlem, The Netherlands). Plates were gown for 3–4 weeks until calli and curled thalli were formed. These tissues were then chopped again into 1 mm pieces and material from one plate was split up into three 100 mL Erlenmeyer flasks each containing 25 mL ½ GB5 medium supplemented with 0.03% L-glutamine (Roth, Karlsruhe, Germany), 0.1% casamino acids (Roth, Karlsruhe, Germany), and 2% sucrose (Roth, Karlsruhe, Germany). 

Transformation experiments were carried out with the pGWB2 vector derivate pMpGWB-mCherry comprising the *hpt* hygromycin resistance gene and mediating mCherry expression [31] under the control of the *M. polymorpha EF1α* promoter, known to confer a strong and almost ubiquitous expression in *Marchantia* [32]. Co-cultivation with *Agrobacterium tumefaciens* strain C58C1 (pGV2260) carrying the pMpGWB-mCherry plasmid was performed as described by Ishizaki et al. [18] with slight modifications according to Althoff et al. [32]. Tissues from the three flasks were transferred after 2 days of co-cultivation to three 50 mL falcon tubes, passively sedimented and the supernatant, containing *Agrobacterium* cells, was replaced by three washing steps with ddH_2_O. Next, plant tissues were divided on nine (three per falcon) square petri dishes (10 × 10 cm) containing ½ GB5 medium supplemented with hygromycin (Roth, Karlsruhe, Germany) (2.5, 5, or 10 µg/mL) and 100 µg/mL cefotaxime (Duchefa, Haarlem, The Netherlands) to remove *Agrobacterium* cells and sealed with micropore tape (3M). 

Selection plates were cultured under long day conditions at 22 °C for 3 weeks. Whole young regenerated transformants were transferred to fresh plates with 10 µg/mL hygromycin and 100 µg/mL cefotaxime selection and mCherry expression was analyzed after 3 weeks of selective growth conditions.

## Figures and Tables

**Table 1:** *R. fluitans* 001TC transformation results. Three independent transformation experiments using the *_pro_*Mp*EF1α*::*mCherry* construct were successively subjected to two hygromycin selection steps with indicated concentrations. Together, 13 T1 lines survived the selection procedure and expressed mCherry fluorescence.

Transformation Experiment (hyg μg/mL)	Plants Surviving 1st Selection	Plants Transferred to 2nd Selection (10 μg/mL hyg)	Plants Surviving 2nd Selection + Expressing mCherry
1 (2.5)	144	144	7
2 (5)	39	39	5
3 (10)	1	1	1

## Supplementary Materials

Supplementary materials can be found at https://www.mdpi.com/1422-0067/21/15/5410/s1.
