# Supplementary material for: Transformation of Riccia fluitans, an Amphibious Liverwort Dynamically Responding to Environmental Changes
Source: Int J Mol Sci. 2020 Jul 29;21(15):5410. doi: 10.3390/ijms21155410 (PMC7432341; doi:10.3390/ijms21155410)
Supplement: Supplementary file 1 [file ijms-21-05410-s001.pdf]

Supplementary Materials:

Table 1. Selection marker analysis for *R. fluitans* 001TC transformation. Chopped LF thalli were cultivated on medium supplemented with indicated concentrations of four antibiotics. - indicates no effect on plants, + mild effect (10–30 % died), ++ severe effect (40–70 % died) and +++ full selection, all plants died. Selected working concentrations are indicated in red.

**hygromycin**	**1 week**	**2 weeks**	**3 weeks**
2.5 µg/mL	+	++	++
5 µg/mL	+	++	+++
10 µg/mL	++	+++	+++
**G418**			
5 µg/mL	−	+	++
10 µg/mL	+	++	++
20 µg/mL	++	+++	+++
**chlorsulfuron**			
0.25 µM	−	−	+
0.5 µM	−	+	+
1 µM	−	+	+
**gentamycin**			
100 µg/mL	−	−	−
200 µg/mL	+	+	+
400 µg/mL	+	+	++

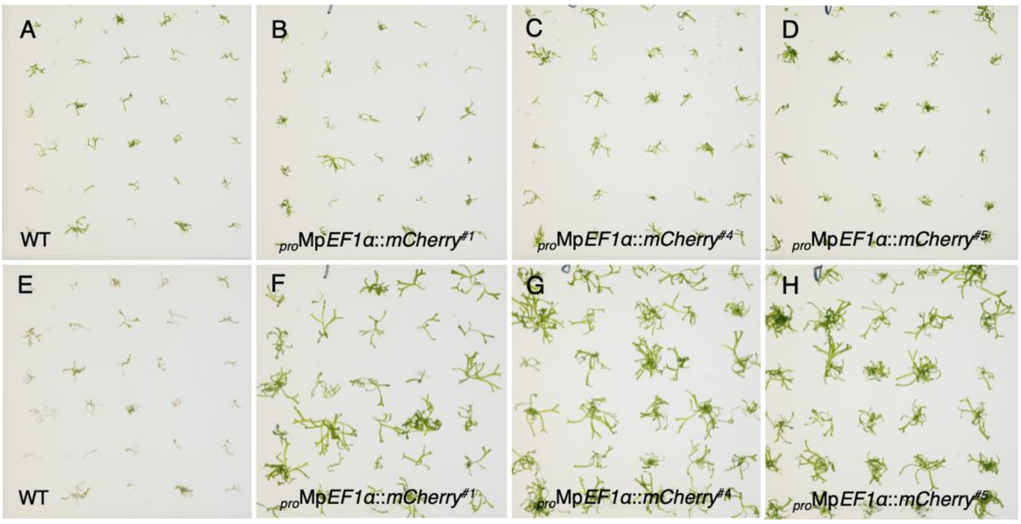

Figure 1. Analysis of transgenic *R. fluitans* 001TC line stability. After three vegetative propagation steps on selection (except for WT), thalli were again chopped and spread on medium without selection. These four-week-old regenerated plants were then transferred from the non-selective medium to medium containing 10 µg/mL hygromycin. **(A)**, **(E)** Wild type. **(B)**, **(F)** $_{pro}$Mp*EF1α*::*mCherry*$^{\#1}$. **(C)**, **(G)** $_{pro}$Mp*EF1α*::*mCherry*$^{\#4}$. **(D)**, **(H)** $_{pro}$Mp*EF1α*::*mCherry*$^{\#5}$. **(A)–(D)** Plates are shown directly after the transfer. **(E)–(H)** 2 weeks after the transfer, wild type plants did not resume growth, whereas all transgenic plants survived and revealed normal growth. 60 plants were transferred on two plates, one plate with 30 plants is shown for each line and time point.

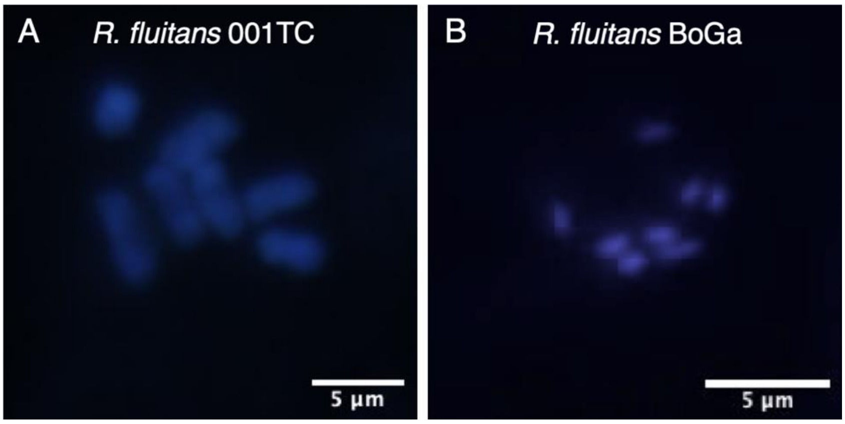

Figure 2. Analysis of *R. fluitans* 001TC and BoGa chromosome numbers by DAPI stainings. **(A)** DAPI staining and successive squash mount of *R. fluitans* 001TC thallus tips. Seven, and not 16 chromosomes are visible, supporting *R. fluitans* species affiliation. *R. fluitans* has one smaller chromosome [54], which is not visible in the image. **(B)** Squash mount of *R. fluitans* BoGa tissue with 8 chromosomes, indicating *R. fluitans* species affiliation. Scale bars 5 µm.
